# Circulating Sphingosine-1-Phosphate as A Non-Invasive Biomarker of Heart Transplant Rejection

**DOI:** 10.1038/s41598-019-50413-8

**Published:** 2019-09-25

**Authors:** Estefanía Tarazón, Carolina Gil-Cayuela, María García Manzanares, Marta Roca, Francisca Lago, José Ramón González-Juanatey, Elena Sánchez-Lacuesta, Luis Martínez-Dolz, Manuel Portolés, Esther Roselló-Lletí

**Affiliations:** 1 Myocardial Dysfunction and Cardiac Transplantation Unit, Health Research Institute Hospital La Fe (IIS La Fe); and CIBERCV, M.P., Avd. Fernando Abril Martorell, 106, 46026 Valencia, Spain; 2 Medicine and Animal Surgery, CEU Cardenal Herrera University, C/Tirant lo Blanc, 7. 46115 Alfara del Patriarca, Valencia, Spain; 3 Analytical Unit, Health Research Institute Hospital La Fe (IIS La Fe), Avd. Fernando Abril Martorell, 106, 46026 Valencia, Spain; 4 Cellular and Molecular Cardiology Research Unit, Department of Cardiology and Institute of Biomedical Research, University Clinical Hospital; and CIBERCV, M.P., Travesía de Choupana, s/n, 15706 Santiago de Compostela, Spain; 5 Hemodynamic Unit, Cardiology Department, University and Polytechnic La Fe Hospital, Avd. Fernando Abril Martorell, 106, 46026 Valencia, Spain; 6 Heart Failure and Transplantation Unit, Cardiology Department, University and Polytechnic La Fe Hospital, Avd. Fernando Abril Martorell, 106, 46026 Valencia, Spain

**Keywords:** Metabolomics, Heart failure

## Abstract

Accumulating evidence has confirmed that the expression of sarcoplasmic reticulum calcium ATPase 2a (SERCA2a) is downregulated in heart failure and cardiac allograft rejection. Although many SERCA2a-related genes and proteins involved in the regulation of myocardial Ca^2+^ fluxes have been explored, its related metabolites remain poorly studied. Our main objective was to identify circulating SERCA2a-related metabolites altered in cardiac allograft rejection and to determine whether these could serve as non-invasive biomarkers. Sixty plasma samples from adult heart transplant were included in a metabolomic analysis. Sphingosine-1 phosphate (S1P), metabolite closely related with SERCA, were increased in patients with cardiac rejection (p < 0.0001). S1P discriminated between patients with and without rejection: normal grafts vs. all rejecting grafts (AUC = 0.911, p < 0.0001), normal grafts vs. Grade 1 R (AUC = 0.819, p < 0.01), Grade 2 R (AUC = 0.911, p < 0.0001), Grade 3 R (AUC = 0.996, p < 0.0001). In addition, we found changes in key enzymes and receptors of S1P pathway analysed on explanted hearts from heart failure patients. This preliminary study reveals that circulating S1P determination could be a novel approach to detect cardiac rejection, showing a robust capability for detection that improves gradually with the severity of rejection. These alterations could be relevant to better understand the involvement of calcium regulation on the pathophysiology of rejection.

## Introduction

Graft rejection is a leading cause of death after transplant^1^. Endomyocardial biopsy (EMB) is the standard rejection screening procedure but presents relevant risks for patients and has technical limitations^2,3^. Although effective alternatives have been studied for years^4–12^, non-invasive monitoring of rejection remains a challenge. Imaging techniques and use of peripheral blood biomarkers have greatly progressed, however some of them have shown poor diagnostic precision or have complex execution. Further studies are needed to establish a simple and non-invasive alternative for detecting heart graft rejection, and reveal the pathophysiologic mechanisms for preventing and treating this condition.

Sarcoplasmic reticulum calcium ATPase 2a (SERCA2a) has received central attention because of its critical role in regulating calcium cycling, being abnormal Ca^2+^ cycling a primary cause of cardiac dysfunction^13^. This molecule is also a relevant target in gene therapy for heart failure. Gene therapy and gene delivery methods have shown significant progress, and the results from important trials confirm the need of further investigation^13–17^. Furthermore, it is involved in cardiac allograft rejection; we previously demonstrated that SERCA2a serum levels change during heart rejection^10^. These findings suggest that SERCA2a concentration assessment may be a relatively simple, capable and non-invasive test for detecting heart transplant rejection. Although many SERCA2a-related genes and proteins involved in the regulation of myocardial Ca^2+^ fluxes have been explored in heart disease^18,19^, few related metabolites have been studied. Specifically, we previously observed partial translocation of nitric oxide by nitric oxide synthase 1 (NOS1) to the sarcolemma in ischemic hearts^19^ and that the upregulation of cardiac NOS1 is not accompanied by increased NOS1 activity in dilated cardiomyopathy^20^. This SERCA2a-related protein may be significant in the pathophysiology of human ischemic and dilated heart disease with a preservative role in maintaining myocardial homeostasis. Given the importance of this pathway, an in-depth understanding of its biologic role is necessary to find potential avenues for a continuous improvement of diagnosis and treatment.

Metabolomics is an emerging science that analyses the metabolome in complex biological systems by combining sensitive analytical techniques^21^. The use of metabolomics in clinical cardiac approaches has the potential to identify new diagnostic and/or prognostic biomarkers for improving the ability to understand pathophysiological pathways related to cardiac allograft rejection. We conducted a metabolomic study with the objective of identifying plasma SERCA2a-related metabolites altered in cardiac allograft rejection. We identified sphingosine-1-phosphate (S1P), a metabolite related with SERCA2a, as a potential non-invasive marker of cardiac rejection, highlighting the involvement of calcium-handling in the rejection process. In addition, we analysed the S1P pathway through the mRNA determination of the key enzymes in the S1P generation and breakdown and also its receptors on explanted hearts from patients with heart failure.

## Results

All groups of post-transplant patients included in the metabolomic study were similar regarding variables such as age, gender, body mass index, diabetes mellitus, dyslipidaemia, echo-Doppler measurements, and hemodynamic parameters. However, we found a higher percentage of patients with hypertension and an increase in lymphocyte number and NT-proBNP and Troponin T levels in the groups with rejection (Table 1).

**Table 1:** Patient characteristics at the time of biopsy and blood sample extraction.

	Non-rejection (n=15)	Rejection (n=45)				p-value
		(all grades combined) (n=45)	Grade 1 R (n=15)	Grade 2 R (n=15)	Grade 3 R (n=15)	
Age, years	53 ± 12	50 ± 12	50 ± 12	51 ± 11	48 ± 14	0.757
Male sex (%)	80	93	93	100	87	0.309
**Indication for cardiac transplantation**						
Ischemic cardiomyopathy (%)	20	41	57	33	33	0.213
Idiopathic dilated cardiomyopathy (%)	60	41	29	33	60	0.168
Other (%)	20	18	14	33	7	0.290
Time between transplantation and study enrolment, months	5.6 ± 3.9	4.2 ± 3.6	5.4 ± 3.9	4.0 ± 3.8	3.3 ± 2.9	0.281
Body mass index (kg/m^2^)	26 ± 5	23 ± 4	24 ± 4	21 ± 3	25 ± 4	0.105
Hypertension (%)	7	46	50	47	40	0.049
Diabetes mellitus (%)	27	50	71	33	47	0.077
Dyslipemia (%)	47	30	50	20	21	0.177
**Echo-Doppler study**						
Ejection fraction (%)	64 ± 9	61 ± 6	57 ± 8	67 ± 6	69 ± 4	0.102
LV end systolic diameter (mm)	27 ± 4	27 ± 4	27 ± 4	28 ± 5	27 ± 3	0.945
LV end diastolic diameter (mm)	44 ± 4	44 ± 4	43 ± 4	45 ± 4	44 ± 3	0.853
**Hemodynamic parameters**						
Mean right atrial pressure (mm Hg)	7.1 ± 3.7	6.8 ± 3.9	6.1 ± 5.0	7.5 ± 3.2	6.9 ± 3.1	0.818
Systolic right ventricular pressure (mm Hg)	33 ± 16	29 ± 16	34 ± 11	36 ± 8	37 ± 8	0.802
Diastolic right ventricular pressure (mm Hg)	7.3 ± 4.1	6.6 ± 3.7	5.8 ± 4.1	6.9 ± 3.3	7.6 ± 4.0	0.730
**Immunosuppressive therapy**						
Tacrolimus (%)	100	100	100	100	100	—
Mycophenolic acid (%)	100	98	100	93	100	0.394
Steroids (%)	100	98	100	93	100	0.394
Neutrophils (thousands/mm^3^)	4.5 ± 2.7	5.6 ± 4.6	4.6 ± 4.0	6.1 ± 4.8	6.3 ± 5.2	0.550
Leukocytes (thousands/mm^3^)	6.6 ± 2.8	8.1 ± 4.7	6.6 ± 3.8	8.4 ± 5.0	9.3 ± 5.2	0.272
Lymphocytes (thousands/mm^3^)	1.4 ± 0.45	1.7 ± 0.8	1.4 ± 0.4	1.5 ± 0.6	2.2 ± 1.1	0.003
Hemoglobin (mg/dL)	11.8 ± 2.3	12.3 ± 1.8	12.5 ± 1.2	12.5 ± 2.0	11.8 ± 2.2	0.658
Hematocrit (%)	37 ± 7	38 ± 5	39 ± 3	39 ± 6	37 ± 6	0.615
NT-proBNP (pg/mL)	200 ± 85	1441 ± 3449	2267 ± 5721	715 ± 519	1266 ± 1246	0.001
Troponin T (ng/L)	13 ± 7	60 ± 98	73 ± 134	62 ± 57	37 ± 26	0.049

LV, left ventricular; NT-proBNP, N-terminal fragment of B-type natriuretic peptide.

**Figure 1:**
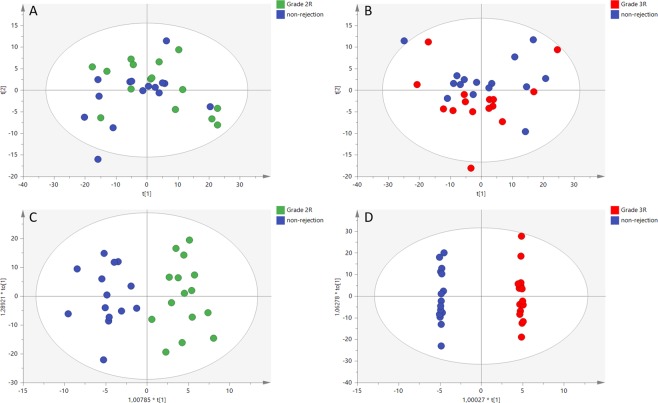
Score plots from PCA and OPLS-DA analysis. PCA score plot (−) ESI mode for normal vs. Grade 2 R (**A**); PCA score plot (−) ESI mode for normal vs. Grade 3 R (**B**); OPLS-DA score plot (−) ESI mode for normal vs. Grade 2 R (**C**); OPLS-DA score plot (−) ESI mode for normal vs. Grade 3 R (**D**).

After data pre-processing in positive and negative ESI modes, overall metabolic differences between non-rejection and rejection groups were evaluated. Better separation between groups was obtained for the negative ionization mode. PCA analysis showed a trend of inter-group separation between the non-rejection group and Grades 2 R and 3 R (Fig. 1A,B). OPLS-DA score plots (Fig. 1C,D) showed a clear discrimination between the non-rejection group and Grades 2 R and 3 R (with Q2Y > 0.5 and CV-ANOVA p < 0.01, in all models). No separation was observed between non-rejection group and Grade 1 R (Supplemental Fig. 1). From the non-rejection group vs. Grades 2 R and 3 R discriminant analysis, we built a variance importance in projection (VIP) plot, summarizing the contribution that each variable makes to the discriminant model. Those variables with VIP > 1 were considered relevant for group discrimination and selected for identification and comparison with those selected in the initial statistical analysis.

As shown in Table 2 we identify thirteen differential metabolites, but only S1P levels showed statistically significant differences between the group of patients without rejection and each group of rejection. Then, we focused on S1P, a metabolite closely related with SERCA2a, as have been reported by other authors’ previously^22,23^. In this study we support this relationship since we have found an inverse significant correlation (r = −0.47, p < 0.0001, Supplemental Fig. 2) between the circulating levels of the S1P and SERCA2a (SERCA2a circulating levels obtained in our previous study)^10^. In addition, we have obtained a significant correlation between S1P levels and lymphocyte number (r = 0.425, p < 0.01).

**Table 2:** Metabolomics identifies differential metabolites between patients without allograft rejection (Grade 0 R) and patients with allograft rejection (Grade 1 R, 2 R, 3 R).

Mass (m/z)	RT (min)	Detection mode	Formula	Adduct	Compound name		p-value		
					Tentative	Confirmed	Grade 0 R vs. 1 R	Grade 0 R vs. 2 R	Grade 0 R vs. 3 R
158.9788	0.53	−	C5H11AsO2	M-H20-H	Arsenobetaine		NS	<0.0001	<0.0001
			C5H5ClN2S	M-H	5-Chloro-3-cyclopropyl-1,2,4-thiadiazole				
			C5H5ClN2S	M-H	2-Chloro-6-(methylsulfanyl)pyrazine				
			C5H5ClN2S	M-H	4-Chloro-6-(methylsulfanyl)pyrimidine				
			C5H5ClN2S	M-H	2-Chloro-4-(methylsulfanyl)pyrimidine				
			C5H5ClN2S	M-H	5-Chloro-2-(methylthio)pyrimidine				
			C5H5ClN2S	M-H	4-Chloro-2-methylthiopyrimidine				
			C5H5ClN2S	M-H	3-Chloro-6-(methylthio)pyridazine				
			C5H5ClN2S	M-H	3-Aminothiophene-2-carbonitrile hydrochloride				
215.0662	0.66	−	C8H14N2O6	M-H20-H	L-beta-aspartyl-l-threonine		NS	<0.05	<0.01
			C8H14N2O6	M-H20-H	Aspartyl-threonine				
			C8H14N2O6	M-H20-H	Threoninyl-aspartate				
234.8864	12.95	+	C3H6S6	M+H	1,2,4,5,7,8-hexathionane		NS	NS	<0.05
			C3H6S6	M+H	1,2,3,5,6,8-Hexathionane				
			C6H3Cl3O	M+K	2,4,6-trichlorophenol				
			C6H3Cl3O	M+K	2,4,5-trichlorophenol				
261.1311	3.35	+	C12H20O6	M+H		Glycerol tripropanoate	NS	NS	<0.05
378.2410	7.94	−	C18H40NO6P	M-H20-H	Phytosphingosine-1-p	Sphingosine 1-phosphate	<0.01	<0.0001	<0.0001
			C18H38NO5P	M-H					
			C18H38NO5P	M-H	N-(1-hydroxy-2-phosphonoethyl)hexadecanimidic acid				
464.3010	6.54	−	C26H43NO6	M-H	Sodium glycocholate		NS	NS	<0.05
			C26H43NO6	M-H	3a,7b,12a-trihydroxyoxocholanyl-glycine				
			C26H43NO6	M-H		Glycocholic acid			
476.2775	8.39	−	C23H44NO7P	M-H		LysoPE(0:0/18:2(9z,12z))	<0.05	NS	<0.01
			C23H44NO7P	M-H		LysoPE(18:2(9z,12z)/0:0)			
478.2929	8.20	−	C23H46NO7P	M-H	PE(18:1(9Z)/0:0)		NS	NS	<0.01
			C23H46NO7P	M-H	Glycerophospho-N-Oleoyl Ethanolamine				
			C24H39N3O4	M+FA-H	(3beta,8xi,9xi,12alpha,14xi)-3-Azido-				
					7,12-dihydroxycholan-24-oic acid				
			C25H41N3O6	M-H	N2-((Benzyloxy)carbonyl)-N6-(tert-butoxycarbonyl)-				
					L-lysine, compound with cyclohexylamine (1:1)				
			C23H46NO7P	M-H	PC(15:1(9Z)/0:0)				
			C23H46NO7P	M-H	PE(18:1(9Z)/0:0)[U]				
			C23H46NO7P	M-H	2,3-Dihydroxypropyl 2-[(octadec-9-enoyl)amino]				
					ethyl hydrogen phosphate				
			C23H46NO7P	M-H		LysoPE(0:0/18:1(9z))			
			C23H46NO7P	M-H		LysoPE(18:1(11z)/0:0)			
			C23H46NO7P	M-H	LysoPE(18:1(9z)/0:0)				
			C23H46NO7P	M-H	LysoPE(18:1(9z)/0:0)				
			C23H46NO7P	M-H		LysoPE(0:0/18:1(11z))			
517.3886	9.45	−	C31H52O3	M+FA-H	Soyasapogenol D		<0.05	NS	<0.05
			C31H52O3	M+FA-H	[2,5,7,8-Tetramethyl-2-(4,8,12-trimethyltridecyl)-				
					3,4-dihydrochromen-6-yl] acetate				
			C32H54O5	M-H	Ganoderiol c				
526.3133	8.36	−	C23H48NO7P	M+FA-H	LysoPC(15:0)		NS	NS	<0.01
			C23H48NO7P	M+FA-H	LysoPE(0:0/18:0)				
			C23H48NO7P	M+FA-H	LysoPE(18:0/0:0)				
			C24H48NO7P	M+FA-H	LysoPC(16:1(9z))				
			C24H48NO7P	M+FA-H	PE(19:1(9z)/0:0)				
			C24H48NO7P	M+FA-H	(2-aminoethoxy)[(2 R)-3-hydroxy-2-[(9Z)-nonadec-				
					9-enoyloxy]propoxy]phosphinic acid				
			C25H50NO9P	M-H	PE(10:0/10:0(3-oh))				
			C25H50NO9P	M-H	PE(10:0(3-oh)/10:0)				
588.3301	8.40	−	C28H50NO7P	M+FA-H	LysoPC(20:4(5z,8z,11z,14z))		NS	NS	<0.05
			C28H50NO7P	M+FA-H	LysoPC(20:4(8z,11z,14z,17z))				
606.3011	8.20	−	C31H41N7O6	M-H		chymostatin	NS	NS	<0.001
			C28H52NO12P	M-H20-H	2-amino-3-{[(2 R)-3-[(3-hydroxydecanoyl)oxy]-2-				
					[(3-hydroxydodecanoyl)oxy]propyl phosphonato]oxy}propanoate				
			C28H52NO12P	M-H20-H	2-amino-3-{[(2 R)-2-[(3-hydroxydecanoyl)oxy]-3-				
					[(3-hydroxydodecanoyl)oxy]propyl phosphonato]oxy}propanoate				
749.5389	10.33	+	C40H77O10P	M+H	PG(18:1(9z)/16:0)		NS	NS	<0.05
			C40H77O10P	M+H	PG(18:1(11z)/16:0)				
			C40H77O10P	M+H	PG(18:0/16:1(9z))				
			C40H77O10P	M+H	PG(16:1(9z)/18:0)				
			C40H77O10P	M+H	PG(16:0/18:1(9z))				
			C40H77O10P	M+H	PG(16:0/18:1(11z))				

RT, retention time.

**Figure 2:**
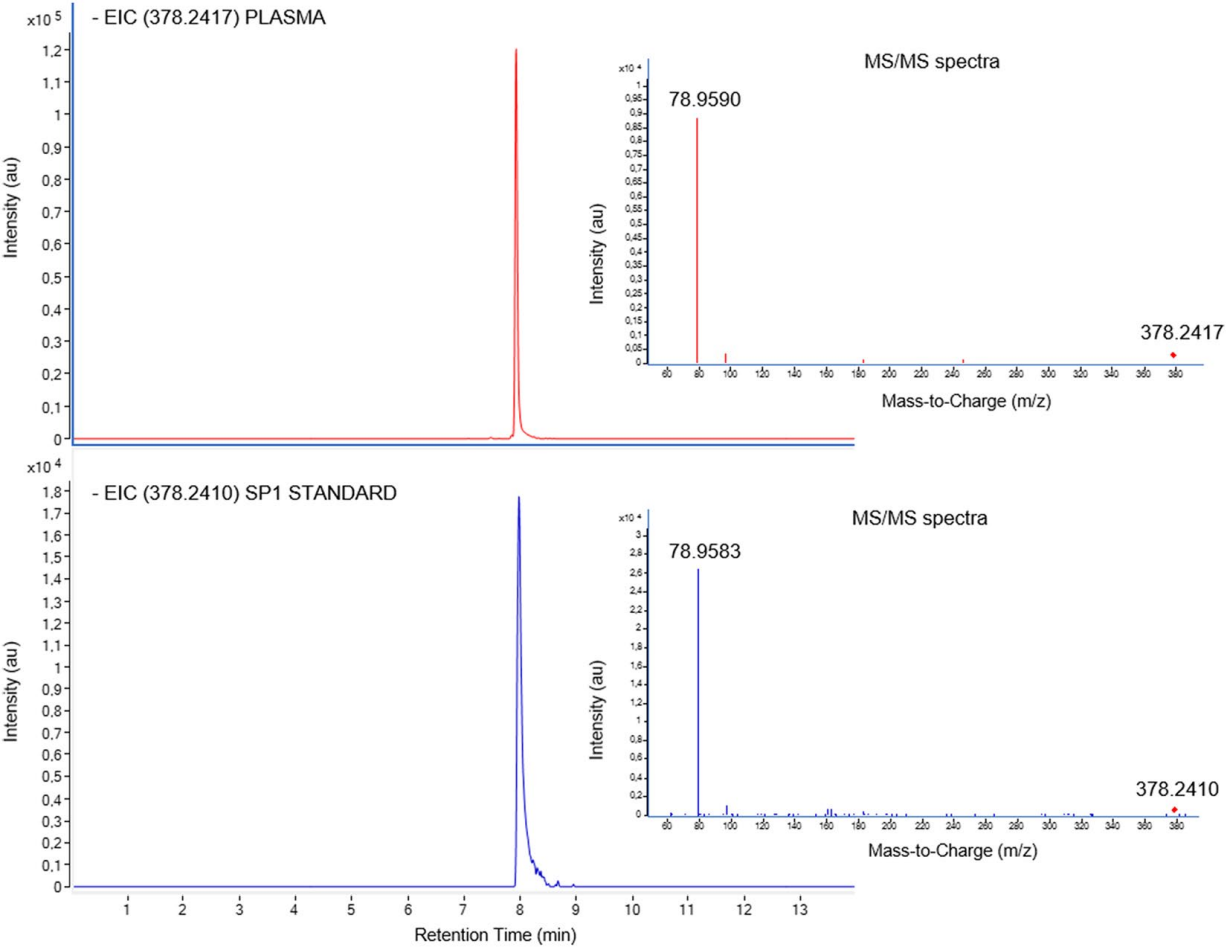
Confirmation of SP1 identity. Comparison of Extracted Ion Chromatogram (EIC) and MS/MS spectra (Collision energy = 20 eV) of SP1 in a plasma sample (m/z = 378.2410) and standard solution (m/z = 378.2410) at 1 ng/mL.

**Figure 3:**
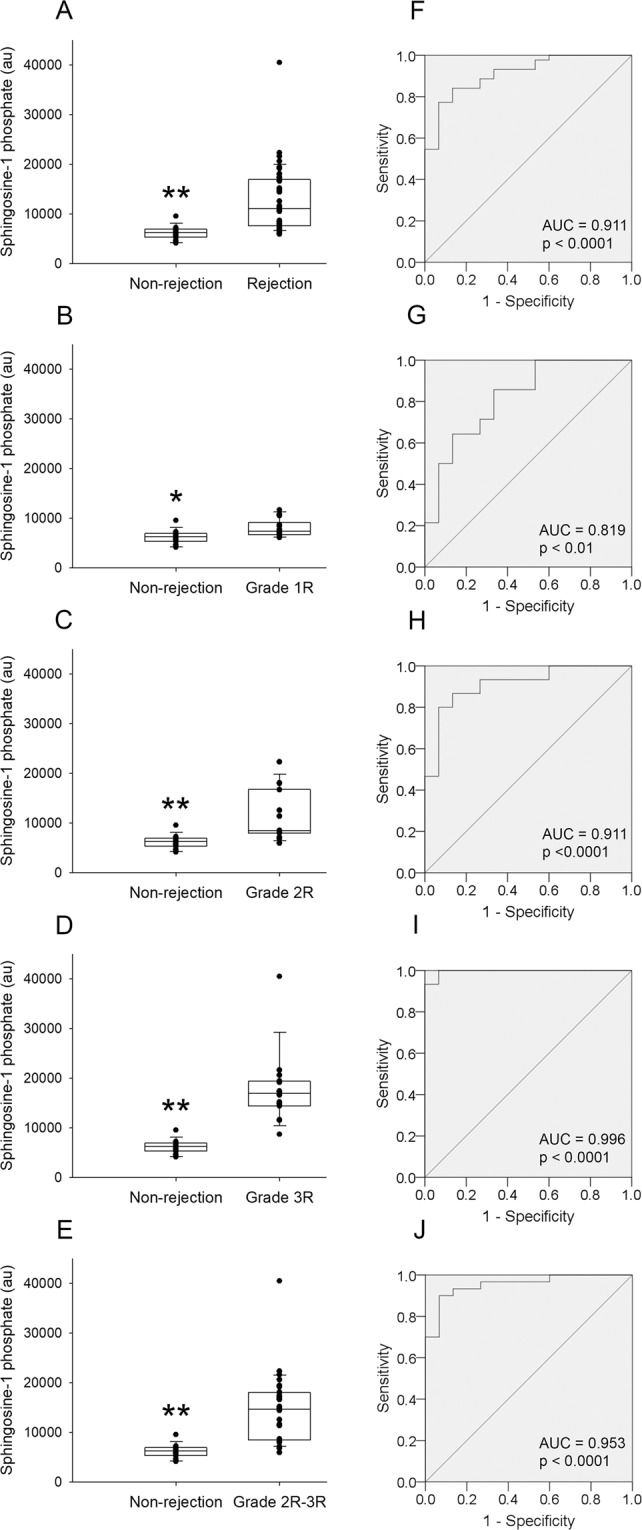
Circulating levels of S1P between non-rejection and rejection heart allografts and receiver-operating characteristic (ROC) curves. Comparison between non-rejection vs. all grades of rejection heart allografts (**A**). Comparison between the different grades of rejection heart allografts (**B**,**C**,**D**). Comparison between non-rejection vs. Grade 2 R and 3 R combined (**E**). The middle line in boxplots represents the median, the lower box bound the first quartile, the upper box bound the third quartile, the whiskers the 95% confidence interval of the fourth mean. ROC curve of circulating S1P for the detection of cardiac allograft rejection: All grades (**F**), Grade 1 R (**G**). Grade 2 R (**H**). Grade 3 R (**I**). Grade 2 R and 3 R combined (**J**). *p < 0.01, ** p < 0.0001.

S1P identity was confirmed by comparing MS/MS spectra and retention time of the chemical standard with plasma samples (Fig. 2). S1P plasma levels were higher in patients with heart transplant rejection (p < 0.0001, Fig. 3A). When we compared patients without allograft rejection with patients of Grade 1 R, 2 R, and 3 R independently, and with 2 R and 3 R combined, we found significant differences for all comparisons (Fig. 3B–E). Additionally, we observed significant differences between Grade 1 R and 2 R (p < 0.05), Grade 1 R and 3 R (p < 0.0001), and Grade 2 R and 3 R (p < 0.01).

ROC curves were performed to analyse the capability of S1P for detecting heart transplant rejection (all grades combined) obtaining a significant area under the curve (0.911 ± 0.040 (95% CI, 0.833–0.988); p < 0.0001) (Fig. 3F). When we divided the heart transplant rejection group into different grades, we observed that S1P detection capability improves in higher grades. For Grade 1 R (0.819 ± 0.078 (95% CI, 0.666–0.972); p < 0.01), for Grade 2 R (0.911 ± 0.054 (95% CI, 0.805–1.000); p < 0.0001), for Grade 3 R (0.996 ± 0.007 (95% CI, 0.981–1.000); p < 0.0001), and for Grade 2 R and 3 R combined (0.953 ± 0.030 (95% CI, 0.895–1.000); p < 0.0001) (Fig. 3G–J). The sensitivity, specificity, positive predictive value, and negative predictive value for the diagnosis of rejection are summarized in Table 3.

**Table 3:** Sensitivities, specificities, and predictive values for the diagnosis of cardiac rejection.

	Cutoff point (au)	Sensitivity (%)	Specificity (%)	PPV (%)	NPV (%)
Grade 1 R	6211	93	47	62	78
Grade 2 R	6780	93	73	73	92
Grade 3 R	10554	93	100	100	94
Grade 1 R, 2 R, and 3 R combined	6613	93	67	89	77
Grade 2 R and 3 R combined	7031	93	87	93	87

PPV, positive predictive value; NPV, negative predictive value.

In light of the relevant results described above, we further investigate on S1P pathway in heart failure. We analysed the mRNA expression of the enzymes involved in S1P synthesis and breakdown, and S1P receptors on cardiac tissue from heart failure patients (Table 4). We obtained a significant decrease in S1P phosphatase 1 (*SGPP1*, p < 0.05) and 2 (*SGPP2*, p < 0.01) levels. S1P receptor 3 levels (*S1PR3*, p <0.05) were also downregulated in patients when we compared with control samples.

**Table 4:** mRNA expression levels of S1P-related genes involved in S1P pathway in heart failure and control hearts.

Gene name	Description	Function	Heart failure hearts (n=26)	Control hearts (n=10)	p-value
*S1PR1*	Sphingosine 1-phosphate receptor 1	S1P receptor activity	480 ± 126	574 ± 393	0.474
*S1PR2*	Sphingosine 1-phosphate receptor 2	S1P receptor activity	1.83 ± 1.86	5.02 ± 8.74	0.271
***S1PR3***	**Sphingosine 1-phosphate receptor 3**	**S1P receptor activity**	**80** ± **40**	**143** ± **77**	**0**.**033**
*S1PR4*	Sphingosine 1-phosphate receptor 4	S1P receptor activity	2.17 ± 2.01	3.60 ± 5.32	0.431
*S1PR5*	Sphingosine 1-phosphate receptor 5	S1P receptor activity	3.18 ± 3.02	2.65 ± 3.46	0.653
*SPHK1*	Sphingosine kinase 1	S1P generation	30 ± 16	36 ± 31	0.562
*SPHK2*	Sphingosine kinase 2	S1P generation	40 ± 16	32 ± 14	0.214
***SGPP1***	**Sphingosine-1-phosphate phosphatase 1**	**S1P dephosphorylation**	**41** ± **14**	**61** ± **39**	**0**.**045**
***SGPP2***	**Sphingosine-1-phosphate phosphatase 2**	**S1P dephosphorylation**	**16** ± **13**	**45** ± **31**	**0**.**016**
*SGPL1*	Sphingosine-1-phosphate lyase 1	S1P breakdown	160 ± 53	172 ± 65	0.586

Data are showed as the mRNA expression levels (arbitrary units) ± standard deviation.

## Discussion

Although the gold standard for the diagnosis of cardiac allograft rejection is the EMB, this procedure may have negative consequences for the patient. Its invasive nature causes patient discomfort and carries the risk of potentially life-threatening complications, including cardiac perforation, tamponade, and arrhythmias^2^. In addition, EMB is subject to sampling error and inter-observer variability^3^. Due to these important risks and limitations, numerous studies have been focused on finding non-invasive monitoring of acute and chronic rejection after cardiac transplantation^4–12^. Overall, these studies demonstrated insufficient diagnostic accuracy, limited sensitivity at lower grades of rejection, limited positive predictive value, and their implementation has been proved complex and in some cases relatively expensive.

This metabolomic study reveals that assessment of plasma S1P allows accurate discrimination between patients with allograft rejection, and those without. We found an increase in S1P levels in peripheral blood in rejection groups, with a capability for detection that improved gradually as rejection grades increased. Our results suggest that S1P has clinical interest as a non-invasive biomarker of heart transplant rejection. Although we have found several phospholipids and other metabolites altered when we compared the different groups of study, we focused on S1P since this sphingolipid is the only altered in the three rejection groups analysed and is also involved in Ca^2+^ regulation.

Sphingolipids such as S1P have been recognized as important second messengers. S1P is a well-known survival factor in many tissues^24^, and has been given substantial attention to obtain additional mechanistic insight into its signalling and biological activity in the cardiovascular system. This metabolite has a greater predictive value in detecting coronary artery disease, than traditional risk factors^25^. In the cardiovascular system, ceramides are catabolized by ceramidase to produce sphingosine which is phosphorylated by Sphingosine kinases 1 and 2 (SPHK1 and SPHK2) to generate the signal transmitter S1P. This molecule is either dephosphorylated by SGPPs and broken down by S1P lyase 1 (SGPL1). When we have evaluated these enzymes on cardiac tissue from heart failure patients we observed a decrease of *SGPPs* mRNA levels. These findings along with the fact of not finding changes in *SPHKs* and *SGPL1* suggest a S1P accumulation in these pathological samples. In fact, previous studies have demonstrated that inhibition of SGPP1 increase S1P accumulation and that SGPP1 overexpression is indicative of reduced S1P signalling^26–28^. S1P is a mediator of ischemic pre- and post-conditioning, with S1P receptors being expressed in the myocardium, endothelium, and platelets^29^. In this study, although we detect mRNA expression of the five S1P receptors in cardiac tissue from heart failure patients, we only observed a significant downregulation in *S1PR3*. In accordance with our results, previous works have shown the S1PR1 and S1PR3 to be the predominant S1P receptor subtype on cardiac tissue^30^.

Interestingly, previous reports support that sphingosine could control the activity of SERCA, hence this sphingolipid participate in the regulation of Ca^2+^ through its inhibitory effect on Ca^2+^- ATPase activity. These authors showed a direct interaction of sphingosine with the Ca^2+^-ATPase^22^. In addition, S1P are reported to trigger endoplasmic reticulum stress^31^ and ceramide disrupts Ca^2+^ homeostasis by inhibiting SERCA expression, thus also increasing endoplasmic reticulum stress^32^. In this sense, our results are in concordance with the previous findings since we have found an inverse correlation between the blood levels of SERCA2a and S1P being altered in opposite direction when the patients present cardiac rejection and finding an inverse relationship between both molecules. Our previous results demonstrated that levels of circulating SERCA2a allow precise discrimination between patients with allograft rejection and those without (Grade 1 R AUC = 0.751, Grade 2 R AUC = 0.875, Grade 3 R AUC = 0.922), being SERCA2a serum levels lower in the rejection groups^10^. Now, we have obtained relevant findings in the same direction, even with an improved capability of rejection. S1P plasma levels were higher in patients with heart transplant rejection and presented a strong rejection detection capability, especially in clinically relevant rejection degrees (Grade 2 R AUC = 0.911, Grade 3 R AUC = 0.996), showing excellent sensitivity, specificity, and positive and negative predictive values. S1P strongly discriminated between patients with allograft rejection Grade 1 R and patients without rejection (AUC = 0.819). In addition, we observed differences in NT-proBNP levels between patients with and without allograft rejection. However, the levels of this peptide did not enable for distinctions between different grades of rejection, suggesting that while NT-proBNP is a factor mirroring an established tissue damage, S1P might change according to damage progression and involvement of vascular component. Taken all together, our findings demonstrate the direct involvement of myocardial calcium homeostasis alterations not only in heart failure condition but also on the physiopathological mechanism leading to cardiac rejection. This disorder is reflected in peripheral blood and discriminates with excellent accuracy between patients with allograft rejection and those without, showing a solid capability for detection that improves in the most clinically relevant rejection degrees.

Improvements towards clinical implementation of new markers are laborious and costly, and meta-analyses and prospective validation are also necessary. In fact, Gene Expression Profiling (Allomap) is the only non-invasive diagnostic test included in International Society for Heart and Lung Transplantation (ISHLT) guidelines to identify the risk of acute cellular rejection in heart transplant recipients. However, it lacks a good positive predictive value and it can only be used to rule out the presence of acute cellular rejection of grade 2 R or greater in appropriate low-risk patients. In addition, AlloMap is specific to acute cellular rejection and cannot diagnose patients with humoral rejection^33^. Consequently, a novel set of biomarkers is greatly required to complement these assays in order to detect organ injury and improve routine clinical practice. New studies focused on to identify circulating microRNAs^8^, serum exosomal proteins^9^, cell-free DNA (cfDNA)^11^ or individual biomarkers^10,12^ as effective and non-invasive diagnostic procedures are emerging with significant results. However, their precise role in mediating rejection remains to be elucidated requiring more work. In addition, pre-transplant assessment of S1P could be necessary to determine its predictive value. The possibility to detect individuals at high risk of early acute rejection before transplantation would make it possible to better surveillance of these patients in order to reduce the risk of late complications.

This metabolomic study reveals that assessment of plasma S1P allows accurate discrimination between patients with allograft rejection, and those without. We found an increase in S1P levels in peripheral blood in rejection groups, with a capability for detection that improved gradually as rejection grades increased. Our results suggest that S1P has clinical interest as a relatively simple, non-invasive biomarker in the diagnosis of heart transplant rejection. However, we show a preliminary study with a limited number of subjects per group that may be validated in larger prospective patient cohorts to contribute to a better investigation on cardiac rejection and lead to the use of this non-invasive determination as an alternative to EMB in the detection of rejection. In this sense, the discovery of precise markers of cardiac rejection like the previously cited, and now S1P as new candidate, could allow the possibility of establishing improved panels for pre- and post-transplantation surveillance.

Our study is limited on several points and the results must be interpreted in this context. Because systemic and myocardial metabolism can change rapidly in response to conditions such as stress or diet, the present study did not address the changes in patient metabolic profiles at different time points. For better data interpretation, the dynamics and stability of metabolomic profiles need to be well-known. Furthermore, our tissue samples are confined to transmural left ventricle apex and we do not discriminate the tissue component such as vessels or lymphocyte infiltration; thus, our findings could not be generalized to all regions of the left ventricle and we cannot specify the precise origin of the alterations found. In addition, this work represents the involvement of one single centre and it is focused on cellular rejection and have not specifically evaluated antibody-mediated rejection, a relevant clinical entity of rejection associated with worse graft survival^34^. However, the current analyses provide valuable information, and these limiting factors could be the goals for future studies.

In conclusion, this preliminary study reveals that circulating S1P determination could be a novel approach to detect cardiac rejection, showing a robust capability for detection that improves gradually with the severity of rejection. The alteration of this SERCA-related metabolite could be relevant to understand better the involvement of calcium regulation on the pathophysiology of transplant rejection.

## Methods

### Collection of samples

The metabolomic study included sixty heart transplant patients (>18 years) from a single centre, University and Polytechnic La Fe Hospital, who were referred for EMB as a scheduled routine screening. Hearts used in the transplants were no procured from prisoners, organs were donated by people who communicated to their families the desire to be donors, and these authorized the removal of organs after death, all of them came from hospitals of the Spanish National Health System. At the time of EMB, blood samples were collected for laboratory analysis. Plasma was separated by centrifugation at 1500xg for 10 minutes at 4 °C, aliquoted, and immediately stored at −80 °C. Study participants were divided into 4 groups: patients transplanted without allograft rejection (Grade 0 R n=15), and 3 subsets of patients with biopsy-proven allograft rejection (Grade 1 R n=15, Grade 2 R n=15, Grade 3 R n=15). In order to compare groups with each other, the same sample size was established for all groups, so that patients were consecutively included according to their Grade until the number of patients per group was equal.

Patients were maintained on a standard immunosuppression regimen, and rejection episodes were assessed according to the International Society for Heart and Lung Transplantation (ISHLT) consensus report^35^. For each sample, we recorded age, gender, body mass index, primary heart disease, interval between transplantation and study enrolment, biochemical markers, echocardiographic parameters, and other clinical characteristics at the time of each biopsy (Table 1). Experimenters were blind to group assignment and outcome assessment, for all experiments.

The study was approved by the Ethics Committee (Biomedical Investigation Ethics Committee of La Fe University Hospital of Valencia, Spain) and was conducted in accordance with the principles outlined in the Declaration of Helsinki^36^. Prior to sample collection, signed informed consent was obtained from each patient.

### Metabolomic analysis

Chemicals reagents, plasma processing and analysis using UPLC-QToF-MS-based untargeted metabolomics, and quality control assurance are included as Supporting Information.

### MS data pre-processing and metabolite identification

Acquired raw files were converted into mzXML format using Proteowizard 3.0 (http://proteowizard. Sourceforge.net/). Data processing was done through our in-house R-script with the XCMS package (https://bioconductor.org/packages/release/bioc/html/xcms.html). The CAMERA package (https://bioconductor.org/packages/release/bioc/html/CAMERA.html) was also used for identification of isotopes and probable adducts. Finally, a data matrix was generated including variables (m/z-retention time), sample ID (observations), and peak intensities. Before statistical analysis, metabolites with a coefficient of variation >30% of their QC values were filtered out.

Metabolite identification of selected variables was performed by query of the exact mass of detected features against the online Human Metabolome Database (HMDB) (http://www.hmdb.ca/) and the Metlin database (https://metlin.scripps.edu) within a specific mass range (±10 ppm). The identity of the metabolites of interest was confirmed by comparing the MS/MS spectra of the selected features with those of the proposed metabolites in the cited online databases. The identity of the selected metabolite was further confirmed by using authentic standards (sphingosine-1-phosphate, Sigma-Aldrich, Madrid, Spain).

### RNA sequencing analysis

We also included an RNA sequencing (RNAseq) study to analyse the main enzymes and receptors of S1P pathway on twenty-six explanted hearts from heart failure patients undergoing cardiac transplantation and and ten age- and gender matched non-diseased donor hearts. Left ventricular samples were collected from near the apex of the left ventricle and maintained in 0.9% NaCl at 4 °C for a maximum of 4.4 ± 3 h after the coronary circulation loss, and then stored at −80 °C until RNA extraction. The appropriate handling and rapid sample collection and storage by our on call (24 h) team, lead to the collection of these high quality samples (RNA Integrity Number (RIN) >9 for all samples). Clinical history, electrocardiogram, and Doppler echocardiography data were available on patients^17,18^. All controls (CNT) had normal left ventricular function (left ventricular ejection fraction >50%), and no history of cardiac disease. CNT samples were obtained from non-diseased donor hearts that had been rejected for cardiac transplantation owing to size or blood type incompatibility. CNT died of either cerebrovascular or motor vehicle accidents.

Methods used for RNA extraction, RNAseq, computational analysis, and gene functional annotation of the RNAseq data were performed as previously described^17,18^. The data presented in this publication have been deposited in the NCBI Gene Expression Omnibus (GEO) and can be retrieved using http://www.ncbi.nlm.nih.gov/geo/query/acc.cgi?acc=GSE55296 (GEO Series accession number GSE55296).

### Statistical analysis

Basal characteristics were expressed as mean ± standard deviation for continuous variables, and percentages for discrete variables. Results for each variable were tested for normality using the Kolmogorov-Smirnov method. Continuous variables not following a normal distribution were compared using the Mann-Whitney test, and categorical clinical variables were compared using the chi-square test. Variables with a normal distribution were compared using the Student’s t-test for continuous variables, and the Fisher’s exact test for discrete variables.

The relativity, sensitivity, specificity, and predictive value of plasma S1P levels for the presence of transplant rejection was assessed by construction of receiver-operating characteristic (ROC) curves. A p <0.05 was considered statistically significant. All statistical analyses were performed using SPSS software (version 20.0; IBM SPSS Inc; Chicago. IL, USA).

Multivariate data analysis (MVDA) of processed data was performed using Simca 14.1 software (Sartorius Stedim Biotech, Aubagne, France). Pareto scaling was used for data scaling. An unsupervised descriptive Principal Component Analysis (PCA) was carried out to provide an overview of the data, observe clustering and trends, and identify strong outliers. Supervised orthogonal-least-squares-discriminant (OPLS-DA) models were used to identify pattern related metabolites, relate metabolic profiles to rejection factors, and confirm patterns observed with PCA. The quality and significance of the supervised models was assessed using the predicted variation in Y (Q2Y) calculated from 7-fold cross-validation, and the CV-ANOVA p-value, both calculated using SIMCA software.

## Supplementary information

ADDITIONAL INFORMATION: MATERIAL AND METHODS, AND RESULTS
