# Supplementary material for: Circulating Sphingosine-1-Phosphate as A Non-Invasive Biomarker of Heart Transplant Rejection
Source: Sci Rep. 2019 Sep 25;9:13880. doi: 10.1038/s41598-019-50413-8 (PMC6761203; doi:10.1038/s41598-019-50413-8)

CIRCULATING SPHINGOSINE-1-PHOSPHATE AS A NON-INVASIVE BIOMARKER OF HEART TRANSPLANT REJECTION

Short title: S1P: NON-INVASIVE BIOMARKER OF CARDIAC REJECTION

Estefanía Tarazón, PhD; Carolina Gil-Cayuela, PhD; María García Manzanares, PhD; Marta Roca, PhD; Francisca Lago, PhD; José Ramón González-Juanatey, MD; Elena Sánchez-Lacuesta, MD; Luis Martínez-Dolz, MD; Manuel Portolés, PhD; Esther Roselló-Lletí, PhD.

SUPPLEMENTARY MATERIAL

MATERIAL AND METHODS

Chemicals Reagents

All LC solvents were of LC-MS grade (Fisher Scientific, Loughborough, U.K.). Formic acid (≥ 95%) was obtained from Sigma-Aldrich (Madrid, Spain). The internal standards used were DL-Phenylalanine-D5 (Cambridge Isotopes Laboratory Inc., Andover, MA, USA), Caffeine-d9 (Toronto Research Chemicals, Toronto, Ontario, Canada), Reserpine (Sigma-Aldrich, Madrid, Spain) and Leucine Enkephaline (Sigma-Aldrich, Madrid, Spain).

Plasma processing and analysis using UPLC-QToF-MS-based untargeted metabolomics

After thawing samples at room temperature, 50 µL of plasma was mixed with 150 µL of ice-cold CH3CN (0.1% HCOOH v/v). Samples were then incubated at -20°C for 30 min for protein precipitation, followed by centrifugation at 13000xg for 10 min at 4°C. 20 µL of the cleared supernatant was transferred to a 96-well plate for LC-MS analysis. 70 µL of H_2O (0.1% v/v HCOOH) and 10 µL of Internal Standard (IS) solution (40 µM) were added to each vial. Quality control (QC) samples were also prepared by mixing 10 µL from each plasma sample.

Metabolomic analysis was performed using an Ultra-Performance Liquid Chromatography (UPLC) system coupled to an iFunnel quadrupole time of flight (QTOF) Agilent 6550 spectrometer (Agilent Technologies, CA, USA). Chromatographic separation was performed using an UPLC BEH C18 (100 × 2.1 mm, 1.7 µm, Waters, Wexford, Ireland). Autosampler and column temperatures were set to 4°C

and 40°C, respectively, and the injection volume was 5 µL. A gradient elution with a run time of 14 min was performed at a flow rate of 400 µL min-1 as follows: 98% of mobile phase A (H_2O, 0.1% v/v HCOOH) was kept for 1 min, followed by a linear gradient from 2% to 15% of mobile phase B (CH3CN, 0.1% v/v HCOOH) in 2 min, from 15% to 50% B in 3 min and from 50% to 95% over subsequent 3 min. 95% B was held for 3 min, followed by a 0.55 min gradient to return to the initial conditions, which were held for 2.5 min for total column recovery. Full scan MS data from 50 to 1700 m/z, with a scan frequency of 6 Hz, were collected. Both positive and negative electrospray ionization modes (ESI +, ESI -) were used and the conditions were set as follows: gas temperature, 200°C; drying gas, 14 L min-1; nebulizer, 60 psi; sheath gas temperature, 350°C; sheath gas flow, 11 L min-1. Automatic MS spectra recalibration was carried out introducing a reference standard into the source via a reference sprayer valve during the analysis.

Quality control assurance

The pooled QC sample was injected (× 5) at the beginning of the batch to condition the analytical platform and intercalated every 7 samples to perform intra-study reproducibility measurements. A blank sample was also prepared to identify background ions associated with the sample extraction and solvents. Sample acquisition was randomized to avoid bias effects of instrument drifts. Sample stability and analytical drift were also investigated through the IS intensities obtained.

RESULTS

Supplemental Figure 1. PCA score plot (-)ESI mode for normal versus Grade 1R.

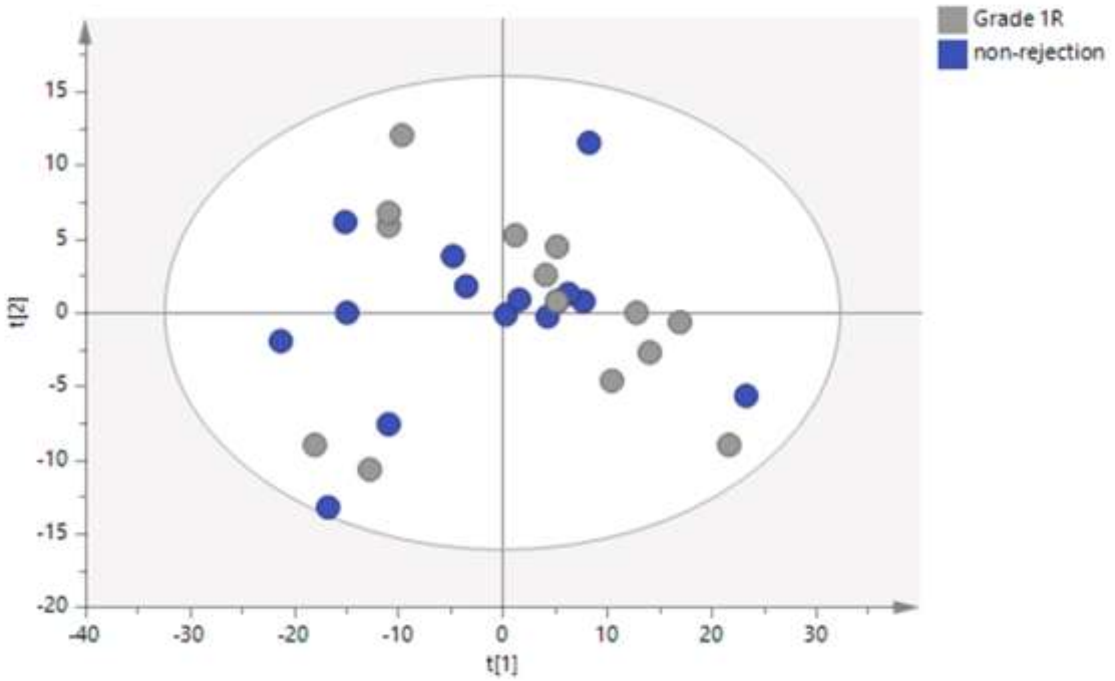

Supplemental Figure 2. Inverse correlation between the circulating levels of the S1P and SERCA2a.

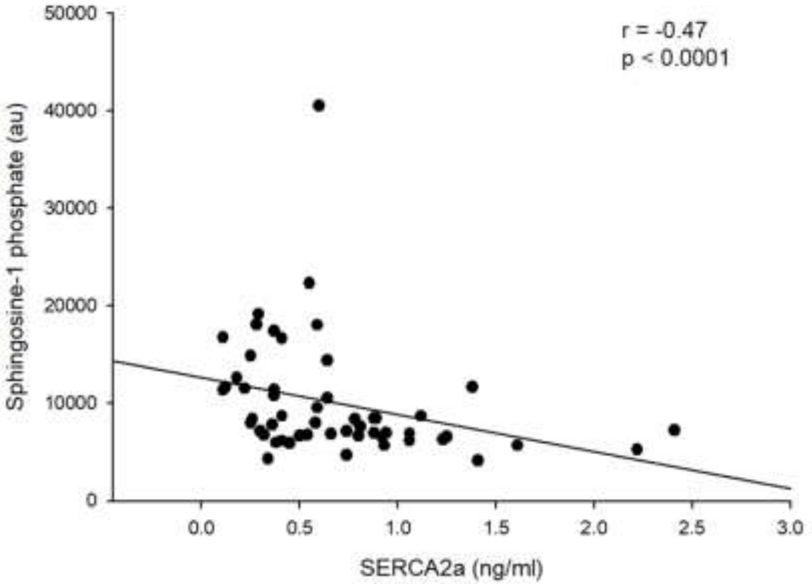

Supplement: Supplementary file 1 — ADDITIONAL INFORMATION: MATERIAL AND METHODS, AND RESULTS [file 41598_2019_50413_MOESM1_ESM.pdf]
